# polyClustR: defining communities of reconciled cancer subtypes with biological and prognostic significance

**DOI:** 10.1186/s12859-018-2204-4

**Published:** 2018-05-25

**Authors:** Katherine Eason, Gift Nyamundanda, Anguraj Sadanandam

**Affiliations:** 1 Division of Molecular Pathology, The Institute of Cancer Research (ICR), London, UK; 2 Centre for Molecular Pathology, Royal Marsden Hospital (RMH), London, UK

**Keywords:** Clustering methods, Subtype community, Reconciliation methods, Network analysis, Hypergeometric test, Breast cancer, Uveal melanoma, Hierarchical clustering, K-means clustering, Non-negative matrix factorization

## Abstract

**Background:**

To ensure cancer patients are stratified towards treatments that are optimally beneficial, it is a priority to define robust molecular subtypes using clustering methods applied to high-dimensional biological data. If each of these methods produces different numbers of clusters for the same data, it is difficult to achieve an optimal solution. Here, we introduce “polyClustR”, a tool that reconciles clusters identified by different methods into subtype “communities” using a hypergeometric test or a measure of relative proportion of common samples.

**Results:**

The polyClustR pipeline was initially tested using a breast cancer dataset to demonstrate how results are compatible with and add to the understanding of this well-characterised cancer. Two uveal melanoma datasets were then utilised to identify and validate novel subtype communities with significant metastasis-free prognostic differences and associations with known chromosomal aberrations.

**Conclusion:**

We demonstrate the value of the polyClustR approach of applying multiple consensus clustering algorithms and systematically reconciling the results in identifying novel subtype communities of two cancer types, which nevertheless are compatible with established understanding of these diseases. An R implementation of the pipeline is available at: https://github.com/syspremed/polyClustR

**Electronic supplementary material:**

The online version of this article (10.1186/s12859-018-2204-4) contains supplementary material, which is available to authorized users.

## Background

Recently, advances in omics technologies have lead to large volumes of data being collected on molecular profiles, including gene expression, in various cancers. Cancers of all types exhibit inter-tumoral (between patient) heterogeneity that can be quantified in part by gene expression. This heterogeneity can help explain the differential prognosis in cancer patients treated with the same therapies. A well-established example is the specific efficacy of trastuzumab (Herceptin) in HER2-positive breast cancer [1]. Previously, we suggested potential differential cetuximab (anti-EGFR therapy) responses in colorectal cancer (CRC) subtypes that we defined previously [2]. More recently, trials of oxaliplatin in Stage II and III CRC found that its effectiveness may be limited to one of these subtypes [2, 3]. In pancreatic cancer, we observed a relatively increased response to gemcitabine in quasi-mesenchymal (QM) subtype cell lines compared to classical subtype cell lines [4]. This result corroborates with the finding by Mofitt et al., that patients with the basal-like pancreatic cancer subtype (equivalent to our QM subtype) have improved response to adjuvant therapy compared to those with the classical pancreatic cancer subtype [5]. Similarly, we showed potential subtype-specific therapies using a panel of breast cancer cell lines and drug response analysis [6]. Nevertheless, for accurate prediction of therapy responses, the challenge lies in defining robust and clinically relevant subtypes.

In breast cancer, where current opinion lies with the existence of 5 intrinsic gene expression subtypes (basal, HER2/ERBB2, luminal A, luminal B, and normal-like), studies have variously reported a number of gene expression subtypes ranging between 4 [7] and 10 [8]. While multiple factors are involved in this apparent discrepancy in defining a number of cancer subtypes, the clustering methodologies employed can significantly contribute to this difference. There are various clustering algorithms that are regularly employed for this purpose, and each has its own strengths according to the underlying structure of the data it is applied to. As clustering algorithms have a huge range of potential applications, selection of the appropriate algorithm to use in any given situation can be difficult. At the same time, the need for the user to inspect the results of each algorithm over a range of numbers of clusters (*k*) and select the optimal solution are often subjective. This situation has been improved by the adoption of various consensus clustering techniques, which allow for visual and quantitative examination of multiple re-runs of the same algorithm so the effects of random starting points can be taken into consideration.

Nevertheless, the use of consensus clustering does not mitigate the effect that algorithm choice has on the clustering solution. The application of different consensus clustering algorithms leads to different numbers of subtypes (numbers of clusters, *k*), and hence, defining the optimal number of clusters is often challenging. This is due to various factors in the design of the algorithm: whether it is ‘greedy’, that is, if it makes the locally optimal choice at each individual stage at the possible expense of finding a global optimum; whether cluster centroids must be located at data points; how iterative algorithms evaluate their convergence to a solution; the “shape” of the discovered clusters; and the metric used to measure sample similarity are some examples [9]. The high dimension of biological data can also demand unreasonable computational time and create a large search domain for the optimal solution. This makes the use of a single algorithm to cluster gene expression profiles, as is often done in subtyping studies, risky. In addition, the clusters found may well be valid, but information about either larger stratification of the data or small but distinct sub-subtypes of low frequency may be lost [10]. It is for this reason that finding methods of reconciling optimal clustering solutions identified by different algorithms is necessary. Cluster reconciliation not only validates the clusters from different algorithms – it can also reveal in greater detail the structure in the data on the macro and the micro scale, from broad classifications resulting from a handful of important functional groups, to rarer and less well-defined sub-subtypes [10, 11].

Here, we demonstrate how to identify optimal solutions and define subtype “communities” by reconciling clusters identified from three different consensus clustering methods - hierarchical clustering (HC) [12, 13], k-means (KM) [14], and non-negative matrix factorization (NMF) [15]. The clusters were further reconciled using at least two approaches. The first, a hypergeometric test to determine the probability that two clusters share the same samples by chance, was previously used to successfully reconcile subtypes of CRC found via clustering in two studies which found three and five optimal subtypes, respectively [2, 10, 16]. It was determined via this analysis that the three subtypes could be appropriately divided into the five sub-subtypes. When four further studies into CRC were published, finding between 3 and 6 optimal clusters [17–20], the Jaccard index was applied to help understand the relationships between these solutions and find “consensus molecular subtypes” (CMS) [11]. The second and a new reconciliation measure used here – calculating the relative proportion of samples in a smaller cluster present in a larger one (termed PMI) – differs from measures of cluster similarity such as the Jaccard index in order to give sub-subtypes a high score, even if they are much smaller than a larger cluster (see Methods section).

All the above reconciliation methods are part of our new framework or package called “polyClustR”. The framework is flexible that other methods can be included any time. Here, we demonstrate how our new pipeline can be used to identify breast cancer gene expression “subtype communities” and to compare with existing intrinsic subtypes [7]. Moreover, we have applied this to uveal melanoma gene expression profiles to define novel gene expression “subtype communities” with different prognosis and chromosomal aberrations associated with them.

## Methods

### Datasets

The breast cancer dataset (Chin, et al.) [21] consists of 118 gene expression profiles of 12,703 genes generated from frozen resected samples. Patients in this cohort were mostly early-stage, and were a mixture of node- and ER-positive and -negative. The discovery uveal melanoma dataset (GSE22138 [22]) consists of gene expression profiles of 42,346 genes for 63 untreated patients, chromosome 3 monosomy status and follow-up metastasis-free survival information. The validation dataset (GSE44295 [23]) contains 58 gene expression profiles (only the tumor samples were considered) for 24,526 genes from enucleation specimens, with metastasis-free survival information [24].

### Finding the optimal number of clusters

It is not optimal for each of the above clustering methods to find a local solution which depends on the initial conditions, rather than a robust clustering that is stable over various input parameters. To address this, we used consensus clustering approaches that repeat several iterations of the same algorithm using different random starting points, and can also perform the clustering over different subsets of samples. Consensus clustering for each algorithm was performed over a range of *k*-values from 2 to 10 and over multiple subsets of the data (80% of samples randomly selected for each of 100 runs). The results of the consensus clustering were then inspected in order to determine the optimal *k*. Determining the optimal *k* from visual inspection alone is subjective, and so quantification of the consensus clustering is required. Here, the cophenetic correlation coefficient [25] and the silhouette width [26] were used to score each clustering. As there is no “gold standard” method for the selection of the value of *k*, the tool user is prompted to choose the number of clusters they wish to use for each algorithm downstream reconciliation based on this output. This allows for flexibility based on the goals of the analysis, as the user can choose a high value of *k* if the objective is to, perhaps, identify novel subgroups of a well-characterised cancer, or to choose a low value of *k* if this solution is more robust.

### Hypergeometric test

Previous work has used the hypergeometric test to determine if different algorithms’ subtypes correspond to one another [10]. In this pipeline, comparisons can be made between any number of clustering algorithms. The hypergeometric test-based false discovery rate (FDR)-adjusted *p*-value indicating the significance of the size of the overlap between two clusters was used.

### Proportion of maximum intersection (PMI)

We introduce the following proportion of maximum intersection, PMI; for clusters *A* and *B*, of arbitrary size:

$$ PMI=\frac{\left(A\cap B\right)}{\min \left\{\left|A\right|,\left|B\right|\right\}} $$

The measure above gives the proportion of samples shared between two clusters out of the maximum possible samples shared (that is, the number of samples in the smaller cluster). If, for example, *A* contains more samples than *B*, PMI is 0 when none of the samples of *B* are in *A* and 1 when all of the samples of *B* are in *A*. This measure has an advantage over alternatives such as the Jaccard index (used in our previous publication [11]) in this context, for the reason that in having different values of *k* clusters from different algorithms will inherently be of different sizes. If the same number of clusters were being compared between algorithms then Jaccard index would give a good measure of cluster overlap. Using our alternative metric gives the same weight to clusters of the same size which are directly analogous between algorithms, as it does to clusters identified via one algorithm which are sub-subtypes of a cluster found by a different algorithm.

### Network community detection

The FDR values produced by the hypergeometric tests and the PMI scores can be represented as edge weights in an undirected network, where vertices are clusters as discovered by the various algorithms. This network can be subjected to community detection, where groups of well-connected vertices (clusters) are identified. In the label propagation method [27] each vertex is initialized with a different label, before then being assigned the label that is most common amongst its direct neighbors. This process continues iteratively until convergence. A variant of the label propagation algorithm [27], which takes into account the weight of edges connecting the vertices, is used here.

### Statistical analysis

FDR values for enrichment of gene sets were reported as calculated by the Broad Institute’s GSEA software [28]. *P*-values generated by hypergeometric tests were FDR-corrected for multiple testing. Kaplan-Meier analysis was used to assess survival and the statistical analysis were from log-rank test. Prediction analysis of microarrays (PAM) analysis to generate centroids and assign subtypes using Pearson correlation and gene expression data was done as previously described [11].

### Software

Code for hierarchical and k-means consensus clustering was adapted from the *ConsensusClusterPlus* v1.36.0 [29] R package. NMF was performed via the *nmf* v0.20.6 R package [30]. The *igraph* R package v1.0.1 [31] was used for plotting networks and community detection. Silhouette width was calculated and plotted using the *silhouette* function from the R package *cluster* v2.0.4 [32]. Survival analysis was performed using the *survival* v2.39–5 R package [33]. The pipeline described in this paper is publicly available on GitHub at https://github.com/syspremed/polyClustR. Clustering parameters for the pipeline are: Consensus resamplings: 100; Proportion of items sampled per subsample: 0.8; Clustering distance: Euclidean; Heirarchical linkage method for subsampling: Average; Heirarchical linkage method for consensus matrix: Average.

## Results and discussion

### An overview of the tool

**Fig. 1:**
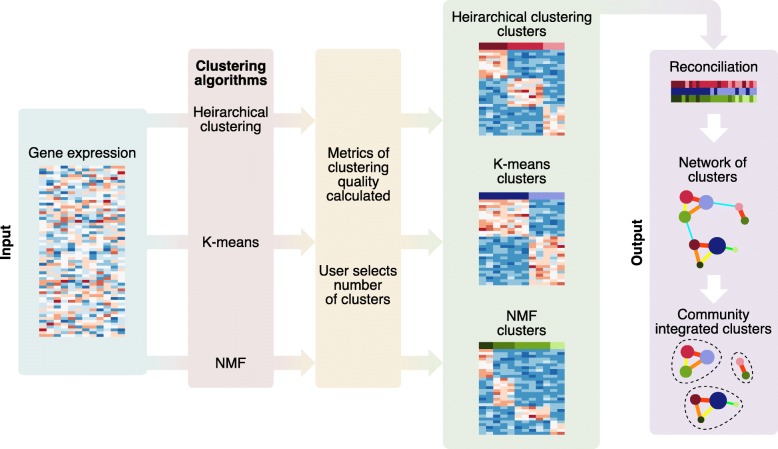
An overview of our pipeline for cluster reconciliation. Gene expression – or other equivalently structured molecular data – is input as a genes by samples matrix. This data is then fed through multiple consensus clustering algorithms (in this case, HC, KM and NMF) to produce multiple clustering solutions. The quality/robustness of the clusters is assessed. These clusters from multiple clustering methods are then reconciled to create “subtype communities” of similar clusters from across the algorithms’ solutions, by applying community detection to networks representing the similarity between clusters from all the algorithms

Our reconciliation method (Fig. 1) uses a matrix of preprocessed and normalized gene expression (or any other similar data) and performs the following: a) applies different consenusus clustering methods (including NMF, HC and KM) and uses statistical scores (specific to each method described below) for each clustering to allow the user to choose the optimal number of clusters; and b) reconciles the results from different clustering methods and identifies a consensus solution by creating network of clusters that defines “communities” of integrated subtypes using methods such as the hypergeometric test and PMI. We then identify the optimal “communities” with highest average silhouette width [26] and compare this reconciliation to known subtypes, if they exist, for that set of samples. To illustrate this, we used published gene expression profiles from breast cancer and uveal melanoma as examples.

### Application to reconcile breast cancer “subtype communities” with intrinsic subtypes

#### Breast cancer subtypes defined by multiple clustering methods

**Fig. 2:**
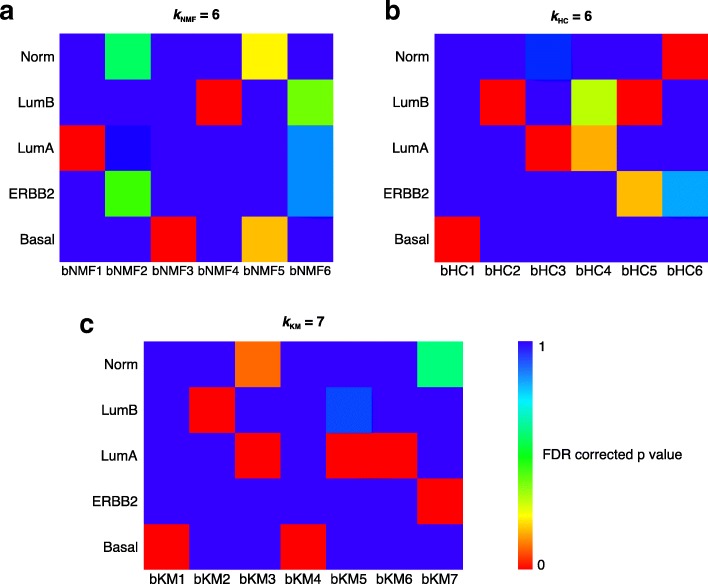
Breast cancer subtypes and their association with intrinsic subtypes – application of polyClustR. **a-c** Heatmaps of similarity of each set of clusters generated by consensus **a** NMF, **b** HC and **c** KM to the known breast cancer subtypes of each sample using 118 breast cancer samples from the published Chin dataset [21] is shown. A hypergeometric test was used to test the significance of overlap between the clusters and the known subtypes. bNMF, bHC and bKM represent NMF, HC and KM breast cancer subtypes, respectively. Norm – normal-like, LumA – luminal A and LumB – luminal B subtypes. FDR – false discovery rate

For this purpose, we used breast tumor gene expression data (*n* = 118; Chin data set) from a published study [21]. Details of initial clustering of this dataset and selection of *k* clusters for each algorithm are provided in Additional file 1: Figure S1A-D. Initially, we applied NMF to the 2258 most highly variable genes from this Chin data set as selected by standard deviation (SD > 0.8). We identified highest cophenetic correlation coefficient of 0.9997 for NMF *k*_*NMF*_ = 2 followed by 0.9962 at *k*_NMF_ = 6. Silhouette width also showed peaks at *k*_NMF_ at 2 and 6 (Additional file 1: Figure S1A-B). In this work, as the downstream reconciliation methods will pool any similar clusters together, we choose the higher value of *k* where the cophenetic correlation coefficient and silhouette width are approximately equal between different values of *k*. In addition, we assessed the consensus matrix for each *k* and evaluated the number of outlier samples (less the better) in each cluster. This allows us to retain the finer stratification of the initial clustering, while simultaneously assembling information about broader biological characteristics from the subsequent reconciliation. As such, we chose *k*_NMF_ = 6*,* and named the clusters breast cancer (b)NMF1 to 6. Overall, known subtypes of these samples [21] were significantly associated with these clusters (Fisher’s exact test; *p* < 0.001). Specifically, the clusters bNMF1, bNMF3 and bNMF4 were significantly associated with luminal A, basal and luminal B, respectively (hypergeometric test; false discovery rate; *FDR* < 0.01) (Fig. 2a). The basal subtype was also border-line significantly associated (*FDR* = 0.2) with bNMF5, suggesting the existence of a sub-subtype of basal breast cancer that was not identified earlier when subtypes for this dataset were predicted by correlation with intrinsic subtype signatures [34] [7]. bNMF2 and bNMF6 were not significantly associated with any of the published subtypes. Gene set enrichment analysis (GSEA) of these unidentified subtypes revealed associations with metaplastic breast cancer (bNMF2, *FDR < 0.01*) and with 17q21-q25 amplicon gene sets (bNMF6, FDR < 0.1) (Additional file 2: Figure S2A-B). Overall, application of NMF to the Chin data set identified clusters that partially overlapped with published subtypes, and others with interesting breast cancer biology.

Since NMF identified extra subtypes in the Chin data set, we applied two additional clustering methods – consensus hierarchical clustering (HC) and k-means (KM). When we applied consensus hierarchical clustering to the same data, *k*_HC_ = 2 and *k*_HC_ = 6 had the highest silhouette widths. (Additional file 1: Figure S1A and C). The cophenetic coefficient after *k*_HC_ = 6 does not increase significantly and the consensus plot showed well-defined clusters (Additional file 1: Figure S1A and C). Hence, we chose six HC clusters (as it reduces the heterogeneity and further downstream reconciliation will group any similar clusters together). The clusters from HC for breast cancer data were defined as breast cancer (b)HC. As with the NMF clusters, these clusters were significantly associated with the known subtypes of these samples (Fisher’s exact test; *FDR* < 0.001). The bHC1, bHC3 and bHC6 clusters were significantly (hypergeometric test; *FDR* < 0.01) associated with basal, luminal A and normal-like subtypes, respectively (Fig. 2b). Both bHC2 and bHC5 were significantly (*FDR* < 0.01) associated with the luminal B subtype. bHC4 was marginally significantly associated with the luminal A subtype, and bHC5 with the ERBB2 (HER2) subtype, with less significance (*FDR* < 0.2; Fig. 2b).

Additionally, we applied consensus KM clustering to the Chin data set. While both the cophenetic coefficient and silhouette width showed highest peaks at *k*_KM_ = 3 and 4 (after *k*_KM_ = 2), we observed that consensus clustering at these *k*_KM_ values did not show clear consensus clusters. There were not large differences in cophenetic coefficient, silhouette width and consensus clusters at *k*_KM_ between 4 and 7 (Additional file 1: Figure S1A and D). Hence, we chose *k*_KM_ = 7 as an optimal cluster. All of these KM clusters (defined as breast cancer (b)KM were significantly or marginally significantly associated with known breast cancer subtypes (Fig. 2c; Fisher’s exact test; *p* < 0.001), unlike the NMF and HC clusters. Specifically, bKM1 and bKM4 were associated with basal, bKM2 with luminal B and bKM3, bKM5 and bKM6 with luminal A (hypergeometric test; *FDR* < 0.01). bKM7 was significantly associated with the ERBB2 subtype, which was not highly significant with any NMF or HC clusters. bKM3 was marginally associated with the normal-like subtype (*FDR* = 0.08). Direct comparison of the two basal clusters through GSEA revealed enrichment of multiple gene sets associated with invasive breast cancer, immunity and cytokines (Additional file 2: Figure S2C-F; with FDR < 0.2). This clearly suggests that different clustering algorithms have the inherent capacity to identify distinct clusters. Here, KM has identified clusters with more significant association to all the published subtypes.

#### Identification of breast cancer “subtype communities”

The existence of multiple clustering solutions defined by different algorithms poses the question of what number of clusters is optimal, and how they reconcile between different methods. To address these questions, we chose two different reconciliation methods – hypergeometric test and PMI. The results from each of the reconciliation methods are discussed below.

**Fig. 3:**
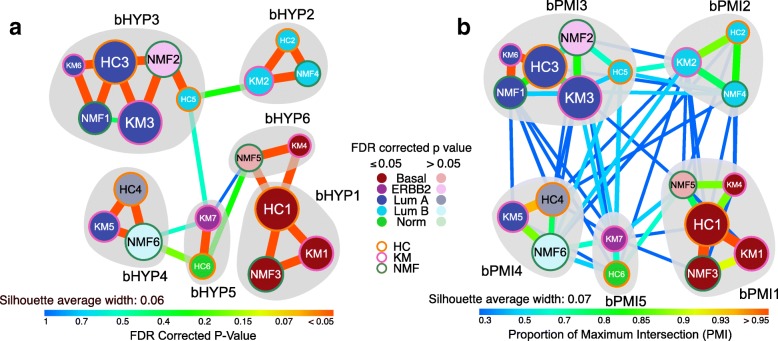
Subtype communities of breast cancer identified using polyClustR. **a** A hypergeometric (HYP) test and **b** PMI was used to assess the significance of the overlap between each pair of clusters using the Chin breast cancer data set [21]. The resulting FDR corrected *p* values/PMI values were plotted as edge colours/weights in this network, with each node representing a cluster. The size of each node represents the number of samples that cluster contains, and those nodes in a lighter shade represent clusters with associations to known subtypes that are not significant (FDR corrected *p* > 0.05). Gray shading marks dense groups of clusters that are defined as subtype communities by network community detection. bHYP and bPMI represent HYP and PMI subtype breast cancer communities, respectively. Average silhouette width is shown for each of the subtype communities. Here, all the HC, KM and NMF represent bHC, bKM and bNMF breast cancer subtypes, respectively

Previously, we have used the hypergeometric test to assess enrichment of samples between two CRC classifications (including ours) as a means of reconciling subtypes [10]. Similarly, we have used this analysis here to reconcile breast cancer clusters between the three different (NMF, HC and KM) algorithms utilized above. Subsequently, in order to group those clusters with significant similarity into “subtype communities”, we performed network community detection by applying weighted label propagation method (using FDR values as edge weights) [27]. As a result, we observed six “subtype communities” (groups of clusters; bHYP1–6) based on this analysis (Fig. 3a).

There was significant association with the known subtypes and these communities (Fisher’s exact test; *p* < 0.001). We observed that five communities were primarily and significantly (hypergeometric test; *FDR* < 0.05) associated with published breast cancer subtypes – bHYP3 and bHYP4 with luminal A, bHYP2 with luminal B, and bHYP1 and bHYP6 with basal (Fig. 3a and Additional file 3: Figure S3A). Four of the communities (bHYP1–4) contained clusters from all three clustering algorithms (Fig. 3a). Interestingly, each of the luminal A and basal subtypes were split into two communities. One basal community (bHYP6) contained the immune-enriched bKM4 cluster. One of the luminal A communities (bHYP3) contained a number of samples from the ERBB2 subtype in a cluster that was enriched for a metaplastic breast cancer signature (bNMF2; Fig. 3a and Additional file 3: Figure S3A), while the other (bHYP4) contained some luminal B samples in the 17q21-q25 amplicon-enriched cluster (bNMF6; Fig. 3a and Additional file 3: Figure S3A). Finally, there was a community (bHYP5) with mixture of normal-like and ERBB2 subtype samples. This community was the most mixed in terms of intrinsic subtypes. Overall, hypergeometric test-based reconciliation expanded the breast cancer subtypes to 6 communities.

Our PMI method is similar to the Jaccard analysis that we used recently to reconcile CRC subtypes as a part of the CRCSC [11], with the difference that it weights sub-groups of a larger cluster as strongly as identical clusters of the same size (see Methods). Here, we applied the PMI method to reconcile subtypes from NMF, HC and KM similar to what we performed using the hypergeometric test. Unlike the hypergeometric method, PMI identified five communities (bPMI1 to 5; Fig. 3b and Additional file 3: Figure S3B), four (bPMI2 to 5) of which were analogous to hypergeometric communities (bHYP2, 3, 4 and 5). The final community (bPMI1) was a combination of the two basal hypergeometric communities (bHYP1 and 6). These communities were significantly associated with known subtypes, overall (Fisher’s exact test; *p* < 0.001). As expected, four of the five communities represent luminal A (bPMI3 and 4), luminal B (bPMI2) and basal (bPMI1) communities (hypergeometric; *FDR* < 0.05). The other community (bPMI5) was a mixture of HER2/ERBB2 and normal-like (Fig. 3b and Additional file 3: Figure S3B).

The merging of the bHYP1 and bHYP6 communities into bPMI1 is due to the large differences in scale of the edges connecting bHC1 to bNMF5/bKM4 and bHC1 to bNMF3/bKM1 in bHYP1 and bHYP6 versus bPMI1 (Fig. 3). FDR-corrected *p*-values between bHC1 and bNMF5/bKM4 were 11–14 orders of magnitude larger than those between bHC1 and bNMF3/bKM1 (despite still being significant). Conversely, the weakest connection to bHC1 in bPMI1 is a PMI value of 0.64, versus 0.77 for bNMF5 to bKM4 and 1.0 for bHC1 to bNMF3 and bHC1 to bKM1 (Fig. 3). This could indicate that the PMI method is able to detect the canonical basal subtype, while the hypergeometric reconciliation is able to find a sub-stratification of this subtype in this breast cancer data set.

To choose the optimal “subtype communities” between HYP and PMI communities, we calculated the silhouette width [26] for all samples in the different communities (Fig. 3 and Additional file 4: Figure S4). The average silhouette widths for HYP communities were 0.06 and that for PMI communities were 0.07. Hence, PMI communities with highest average silhouette width were chosen as optimal.

This application of the pipeline to a well-characterised cancer has demonstrated its ability to identify new biologically distinct “subtype communities” of patients, alongside those subtypes which have already been extensively described. We next sought to apply this pipeline to a cancer with molecular subtypes that have not been explored so comprehensively, uveal melanoma – although classes at the gene expression level are known [35–37].

### Application to uveal melanoma and identification of novel “subtype communities”

#### Identification of subtype communities

Compared to breast cancer, uveal melanoma is a cancer type that has not been extensively subtyped, presumably due to its low incidence. This scarcity of samples makes clustering a challenge – clusters discovered are less likely to be robust due to their small size. It is in cases such as this where the reconciliation of clusters from multiple algorithms may present benefits in terms of increasing confidence in the results of clustering.

As with the breast cancer data, we applied the three clustering algorithms of HC, KM and NMF to a dataset of the 6146 most variable genes (SD > 0.8) from 58 patients with uveal melanoma (GSE22138, [22]). By performing the same assessment of cophenetic correlation coefficient, silhouette width and consensus matrices, we discovered four clusters by HC, six clusters by KM and five clusters by NMF (Additional file 5: Figure S5A-D).

**Fig. 4:**
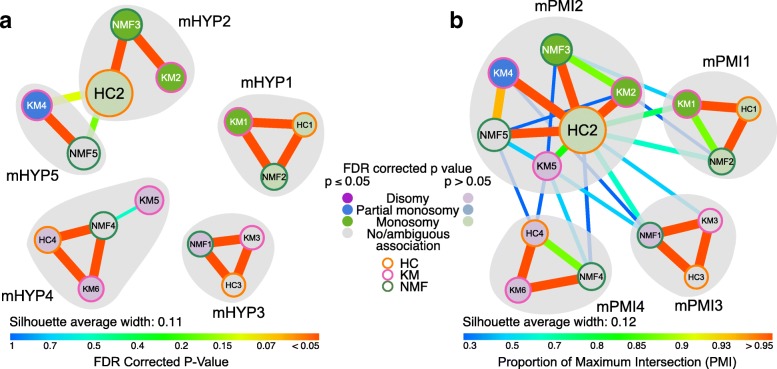
Subtype communities of uveal melanoma identified using polyClustR. **a** A hypergeometric (HYP) test and **b** PMI was used to assess the overlap between each pair of clusters using the uveal melanoma dataset [22]. The resulting FDR corrected *p* values were plotted as edge colours/weights in this network, with each node representing a cluster. The size of each node represents the number of samples that cluster contains, and those nodes in a lighter shade represent clusters with associations to known subtypes that are not significant (FDR corrected *p* > 0.05). Gray shading marks dense groups of clusters as defined by network community detection. mHYP and mPMI represent HYP and PMI subtype melanoma communities, respectively. Average silhouette width is shown for each of the subtype communities

By reconciling these subtypes by a hypergeometric test followed by community detection, we identified five “subtype communities” of clusters (Fig. 4a). When we assessed these communities for the key molecular feature of chromosome 3 aneuploidy, we discovered a significant association of these communities with this feature (Fisher’s exact test; *p* < 0.001); one community – melanoma mHYP2 – was significantly enriched (hypergeometric test; *FDR* < 0.001) for monosomy, and another (mHYP5) was significantly enriched (*FDR* < 0.05) for both disomy and partial monosomy (Fig. 4a and Additional file 6: Figure S6A). Two of the remaining three communities showed less significant associations with chromosome 3 disomy (mHYP4) and monosomy (mHYP1; hypergeometric test; *FDR* < 0.2) respectively, while the final community (mHYP3) was not significantly enriched for either. A similar pattern of associations was observed when assessing four “subtype communities” defined by the PMI method (Fig. 4b), with one community each representing monosomy and disomy (mPMI1 and mPMI4, respectively), and one mixed disomy/partial monosomy/monosomy community (mPMI2) – however the association was not statistically significant (Fisher’s exact test; *p* = 0.6) (Fig. 4b and Additional file 6: Figure S6B). HYP subtypes were chosen over PMI subtypes for significant association with known key molecular features of uveal melanoma and having a lower number of samples with negative silhouette width in this cohort (Additional file 7: Figure S7).

#### Biological understanding of uveal melanoma subtype communities

**Fig. 5:**
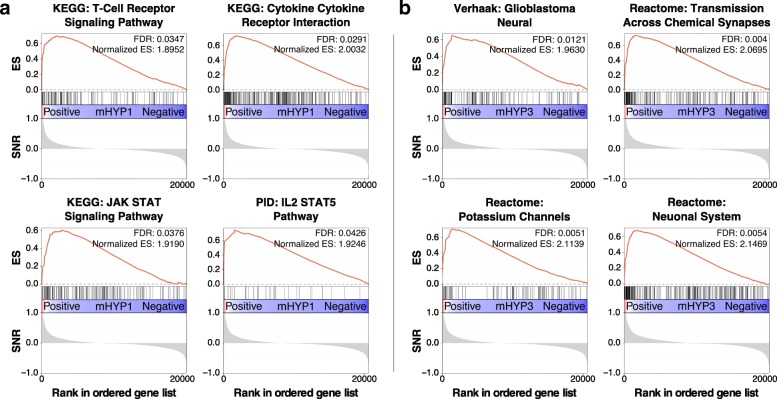
GSEA enrichment plots of **a** the mHYP1 uveal melanoma community, showing significant enrichment of immunity-related gene sets, and **b** the mHYP3 uveal melanoma community, showing significant enrichment of neural-related gene sets. SNR - signal to noise ratio

Next, we sought to understand these communities by performing GSEA, and discovered that one of these communities (mHYP1) was significantly enriched (*FDR <* 0.05) for gene sets associated with immune pathways (e.g. T cell receptor signaling, JAK-STAT, cytokine-cytokine receptor interactions, and IL2 STAT5 pathways; *FDR < 0.05*; Fig. 5a). On the other hand, another subtype (mHYP3) was associated with neural cell types (e.g. glioblastoma neural subtype, neurotransmitter signaling, potassium signaling and neuron system; Fig. 5b; *FDR* < 0.05). The last communities (mHYP2, mHYP4 and mHYP5) did not significantly associate with any gene sets. This could indicate that mHYP2 (which is enriched for chromosome 3 monosomy) and mHYP4 (which is borderline enriched for chromosome 3 disomy) may be defined by their karyotype as opposed to a coherent transcriptomic pattern.

#### Patient prognostic differences between uveal melanoma subtype communities

**Fig. 6:**
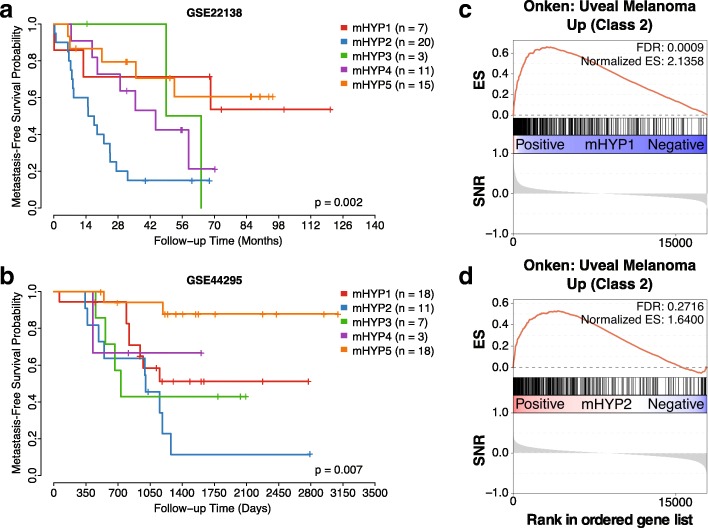
Prognosis and GSEA analysis of uveal melanoma subtype communities. **a**-**b** Metastasis-free survival in the **a** discovery and **b** validation cohorts, respectively, was significantly different between communities. **c-d** GSEA enrichment plots of **c** the mHYP1 and **b** the mHYP2 uveal melanoma communities, showing significant enrichment of class 2 published subtypes. SNR - signal to noise ratio

Since more than 50% of uveal melanoma patients undergo metastasis [22], we assessed the metastasis-free prognosis of the uveal melanoma subtype communities using the GSE22138 [22] data set. Among the two highly frequent communities, mHYP2 (36%) showed significantly poorer metastasis-free prognosis, whereas mHYP5 (27%) showed better prognosis. Both mHYP4 (20%) and mHYP1 (13%) communities showed intermediate prognosis (Fig. 6a).

#### Validation of uveal melanoma subtype communities

Due to the low frequency of some of these communities in this dataset (5% mHYP3, 13% mHYP1), we sought to validate them in an independent dataset consisting of 58 patients with uveal melanoma (GSE44295). Patients were assigned to subtypes based on the correlation of their gene expression profile with the prediction analysis of microarrays (PAM) [38] centroids of each community. 57 samples had metastasis-free survival information. In the validation cohort, 32% of patients were assigned to the mHYP1 (immune-enriched) group, 19% mHYP2 (monosomy-enriched), 12% mHYP3 (neural-enriched), 5% mHYP4 (undetermined) and 32% mHYP5 (disomy/partial monosomy-enriched). In terms of prognosis, these groups showed statistically significant differential metastasis-free survival (*p* = 0.007; Fig. 6b). Analogous to the previous dataset, mHYP2 and mHYP5 communities showed poor and good prognosis, respectively. While mHYP1 showed intermediate prognosis, mHYP4 couldn’t be assessed due to low sample size of only 5% (*n* = 3). Interestingly and similar to the training dataset (GSE22138), 82% of mHYP2 (monosomy-enriched) group in the validation cohort underwent metastasis during follow-up, compared to only 11% of the mHYP5 (disomy/partial monosomy-enriched) group patients. In addition, 33% of intermediate prognostic mHYP4 (undetermined) and 44% mixed prognostic mHYP1 (immune-enriched) patients experienced metastasis. With the increased sample size of mHYP3 (neural-enriched) community, we observed that it has poor overall survival and 57% of the mHYP3 samples underwent metastasis (Fig. 6b). Overall, this identifies and validates novel uveal melanoma subtype communities and their prognostic significance.

#### Comparison of subtype communities to known uveal melanoma classes

Previously, transcriptomic subtypes of uveal melanoma have been defined by clustering of gene expression profiles. Two classes were discovered – class 1, with good prognosis and association with chromosome 3 disomy; and class 2, with poor prognosis, associated with chromosome 3 monosomy and metastasis [35–37]. To reconcile these communities with the gene expression subtypes, we checked for gene set enrichment of the gene signatures for class 1 and class 2 uveal melanomas in this cohort [36]. The class 2 signature was enriched and borderline enriched in the mHYP1 community (immune-enriched; FDR < 0.001; Fig. 6c) and mHYP2 (monosomy; FDR = 0.3; Fig. 6d) groups, respectively, whereas, unexpectedly, the class 1 signature was not significantly enriched in any other group. This could indicate that the class 1 signature defines a heterogeneous set of patients who are not confined to any of our given communities. Overall, this analysis suggests that our novel uveal melanoma subtype communities reveal additional heterogeneity with clinical significance that requires further investigation.

## Conclusions

These results demonstrate that no one clustering algorithm may be relied on to produce clusters which are robust and capture all heterogeneity in a dataset. Instead, multiple algorithms may be applied to the same dataset, and their results compared and reconciled. Our polyClustR tool provides a straightforward interface to cluster datasets using multiple algorithms, provides statistics on the quality of each clustering, and allows the user to fully understand how each result is related through multiple reconciliations. The demonstration that some low-frequency clusters – which may be lost or discarded as outliers if only one algorithm is applied – are consistently identified across algorithms lends credence to their validity, and here such communities were additionally validated in an independent dataset. Thus, the reconciliation of multiple clustering results enables finer stratification of patients’ molecular profiles enabling more focused biological profiling.

## Additional files

Additional file 1: **Figure S1.** Evaluation of consensus clustering of the breast cancer dataset from *k* = 2 to *k* = 10. (A) Cophenetic correlation coefficient (upper) and silhouette width (lower) of the clustering generated by each algorithm for each of *k* clusters. (B) Consensus matrices for NMF clustering. Colors towards red indicate high consensus between runs and those towards blue indicate low consensus. (C-D) Consensus matrices for C) hierarchical clustering and D) k-means clustering. Blue indicates high consensus between different clustering runs and white indicates low consensus. (PDF 472 kb)

Additional file 2: **Figure S2.** Gene Set Enrichment Analysis (GSEA) analysis between subtypes in breast cancer. (A-F) GSEA analysis between the A) bNMF2 and B) bNMF6 breast cancer clusters, showing gene enrichment of metaplastic breast cancer and 17q21–25 amplicon signatures and C-F) between the two basal-subtype (bKM1 and bKM4) k-means clusters, showing enrichment of invasive and immune-related gene sets in bKM4 cluster. (PDF 119 kb)

Additional file 3: **Figure S3.** Comparison of community classifications from each reconciliation method with intrinsic breast cancer subtypes. (A-B) Heatmap showing hypergeometric test with overlap between the subtype communities (from polyClustR) and the known subtypes from A) hypergeometric and B) PMI reconciliation methods. Norm – normal-like subtype; LumA – luminal A subype; Lum B – luminal B subtype. (PDF 50 kb)

Additional file 4: **Figure S4.** Silhouette width of each sample and community in breast cancer for each reconciliation method –hypergeometric (HYP; left) and PMI (right). Colors represent distinct subtype communities. (PDF 21 kb)

Additional file 5: **Figure S5.** Evaluation of consensus clustering of the uveal melanoma dataset from *k* = 2 to *k* = 10. (A) Cophenetic correlation coefficient (upper) and silhouette width (lower) of the clustering generated by each algorithm for each of *k* clusters. (B) Consensus matrices for NMF clustering. Colors towards red indicate high consensus between different clustering runs and those towards blue indicate low consensus. (C-D) Consensus matrices for C) hierarchical clustering and D) k-means clustering. Blue indicates high consensus between clustering runs and white indicates low consensus. (PDF 467 kb)

Additional file 6: **Figure S6.** Comparison of community classifications from each reconciliation method with known chromosome 3 ploidy statuses in uveal melanoma. (A-B) Heatmap showing hypergeometric test with overlap between the subtype communities (from polyClustR) and the known ploidy status from A) hypergeometric and B) PMI reconciliation methods. (PDF 53 kb)

Additional file 7: **Figure S7.** Silhouette width of each sample and community in uveal melanoma for each reconciliation method – hypergeometric (HYP; left) and PMI (right). Colors represent distinct subtype communities. (PDF 19 kb)

## Abbreviations

CMS: Consensus molecular subtypes
CRC: Colorectal cancer
CRCSC: Colorectal cancer subtyping consortium
ER: Estrogen receptor
FDR: False discovery rate
GSEA: Gene set enrichment analysis
HC: Hierarchical clustering
KM: K-means (clustering)
NMF: Non-negative matrix factorisation (clustering)
PAM: Prediction analysis of microarrays
PMI: Proportion of maximum intersection
SD: Standard deviation
