# Supplementary figures and images for: polyClustR: defining communities of reconciled cancer subtypes with biological and prognostic significance
Source: BMC Bioinformatics. 2018 May 25;19:182. doi: 10.1186/s12859-018-2204-4 (PMC5970540; doi:10.1186/s12859-018-2204-4)

Figure S1

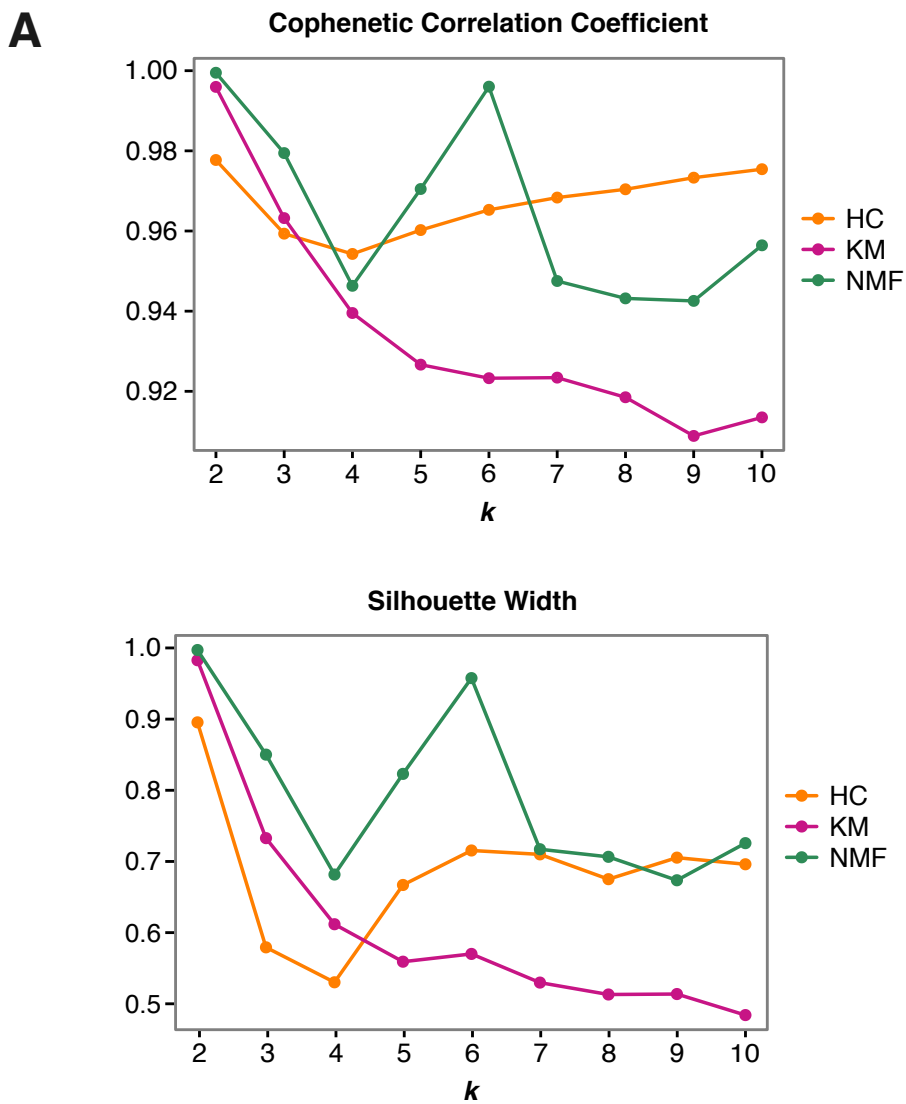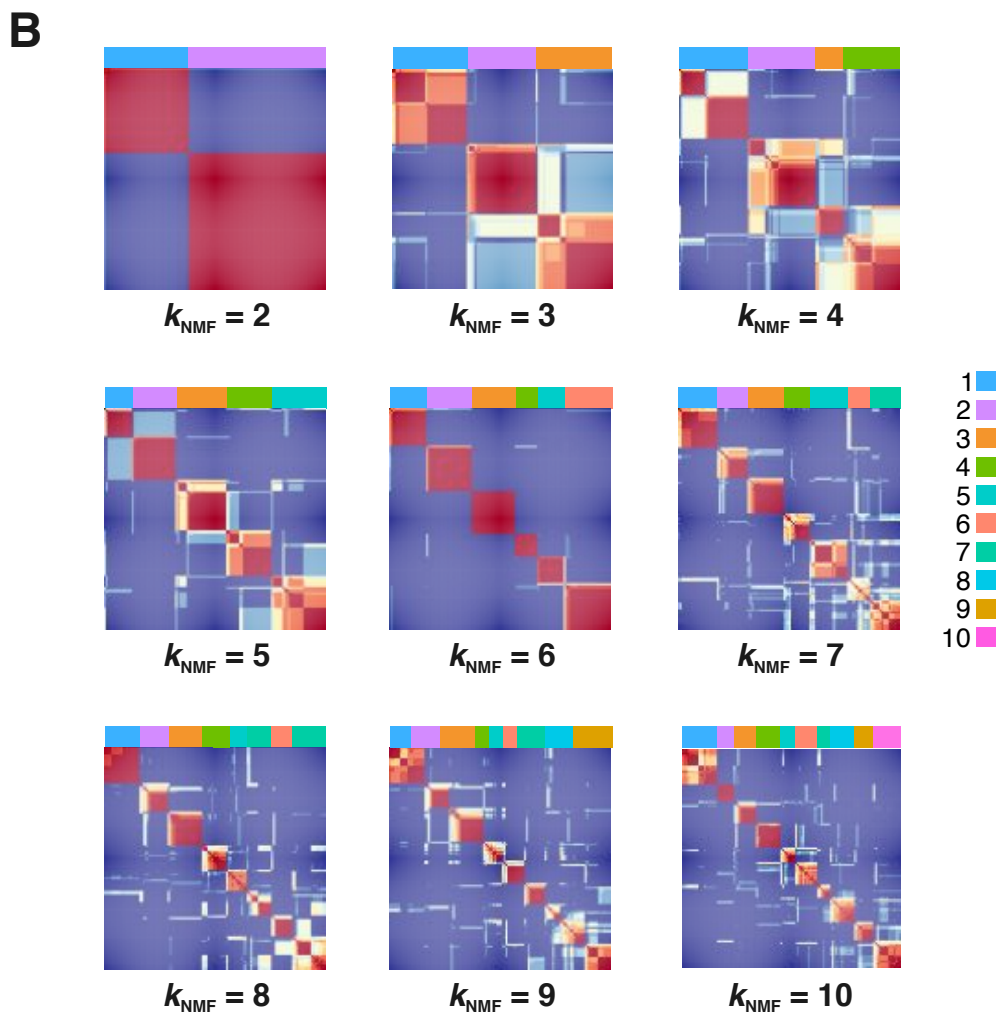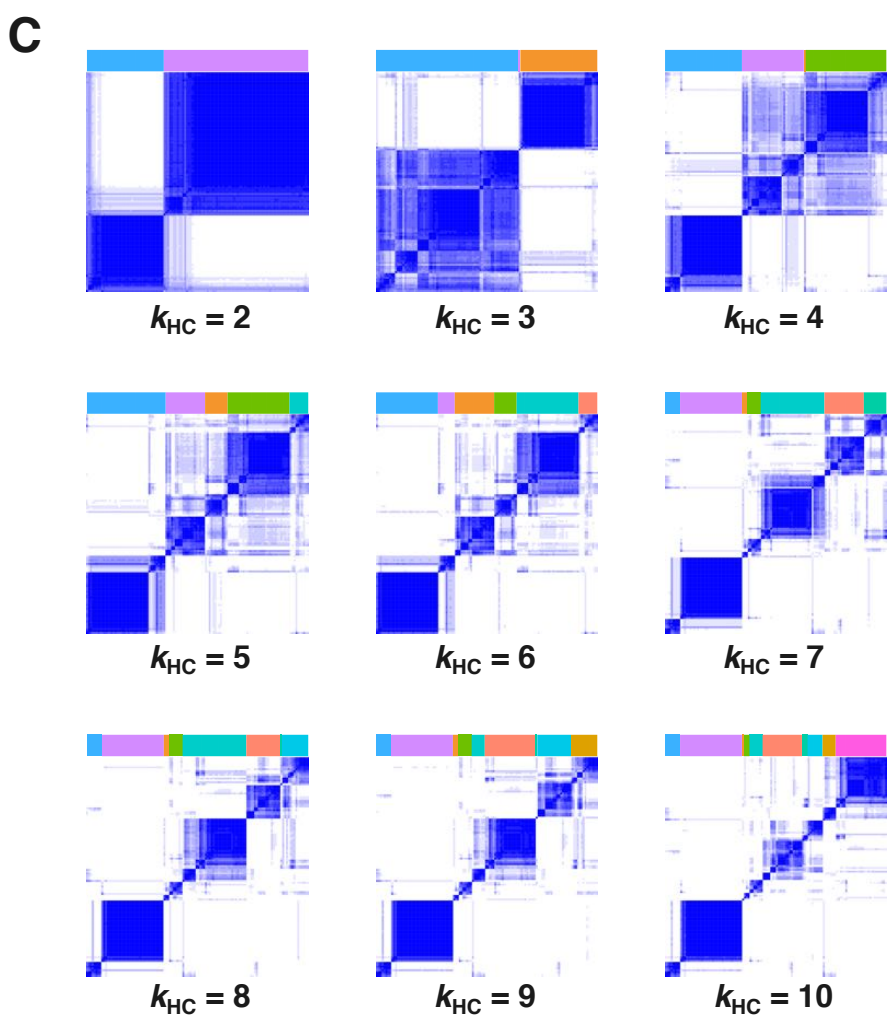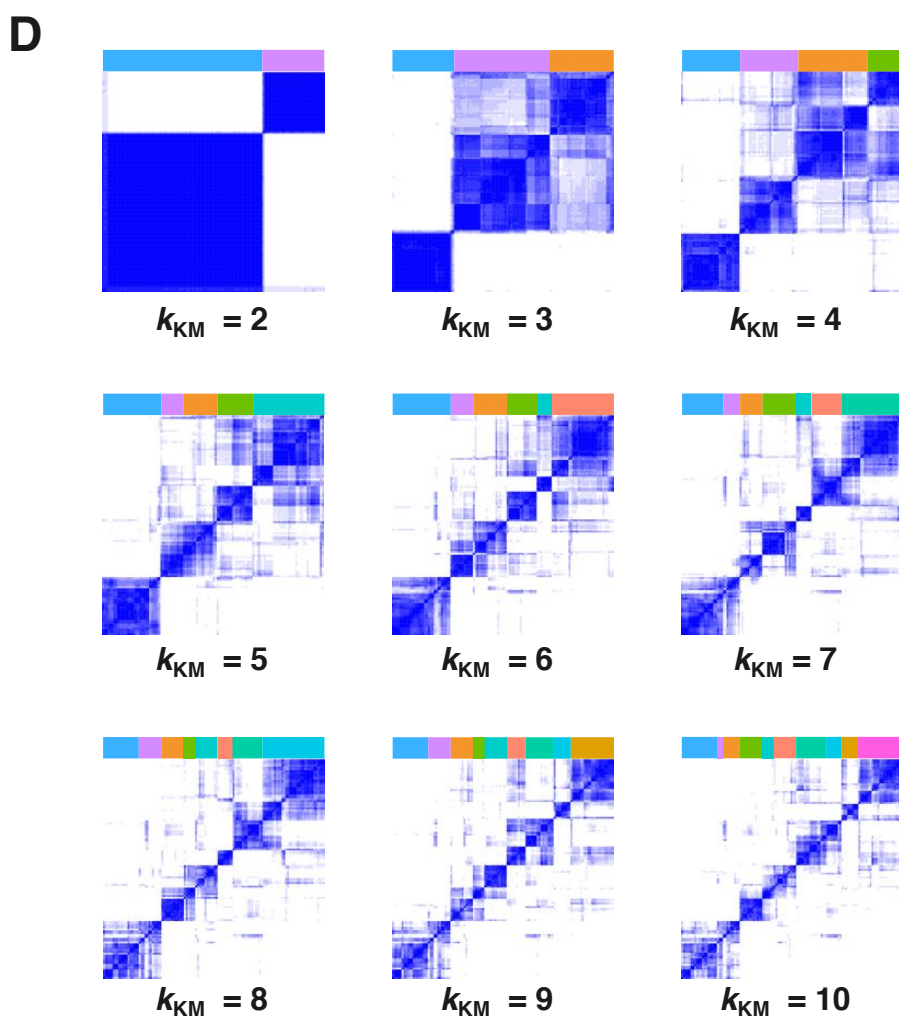

Supplement: Supplementary file 1 — Figure S1. Evaluation of consensus clustering of the breast cancer dataset from k = 2 to k = 10. (A) Cophenetic correlation coefficient (upper) and silhouette width (lower) of the clustering generated by each algorithm for each of k clusters. (B) Consensus matrices for NMF clustering. Colors towards red indicate high consensus between runs and those towards blue indicate low consensus. (C-D) Consensus matrices for C) hierarchical clustering and D) k-means clustering. Blue indicates high consensus between different clustering runs and white indicates low consensus. (PDF 472 kb) [file 12859_2018_2204_MOESM1_ESM.pdf]

Figure S2

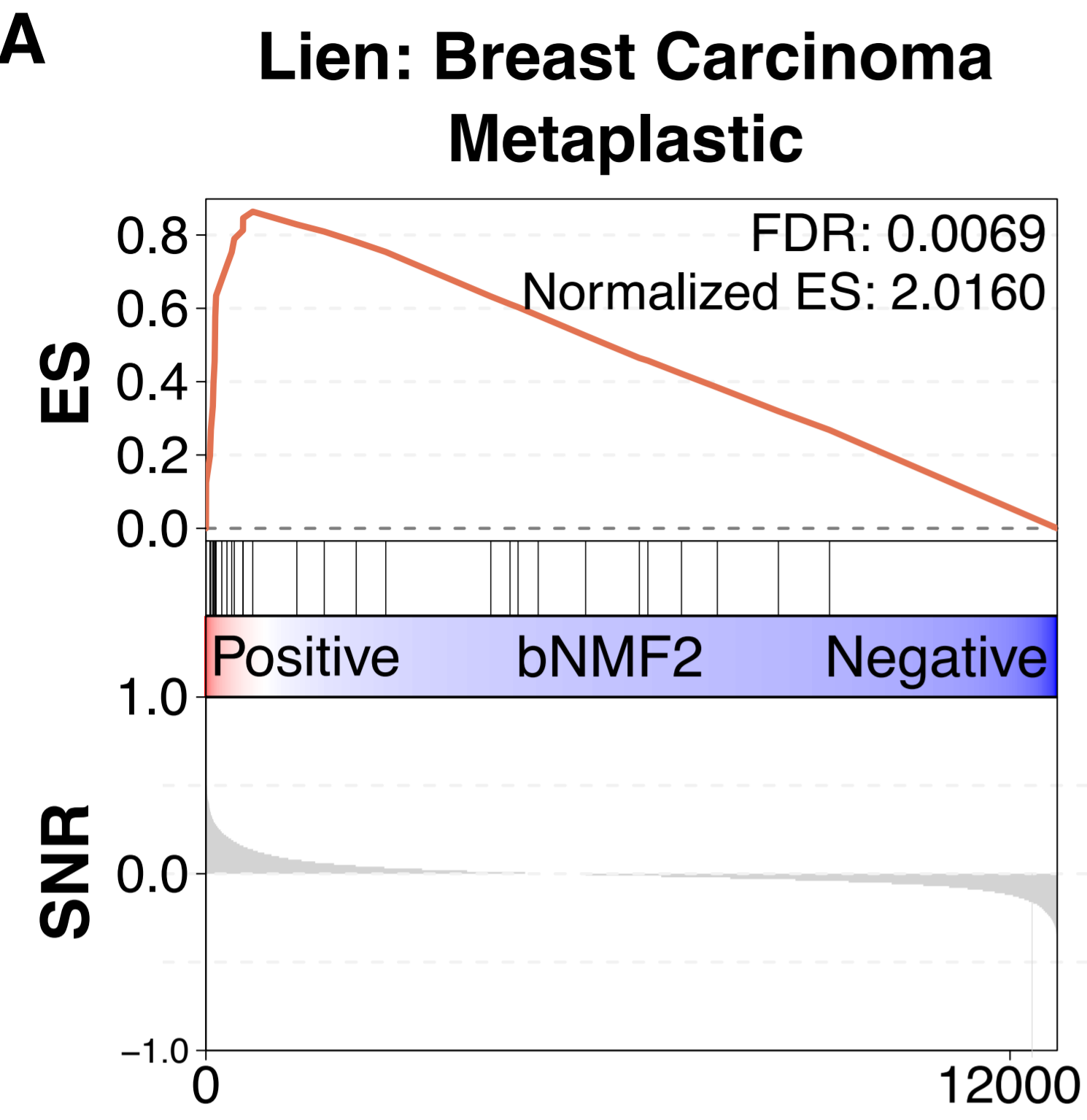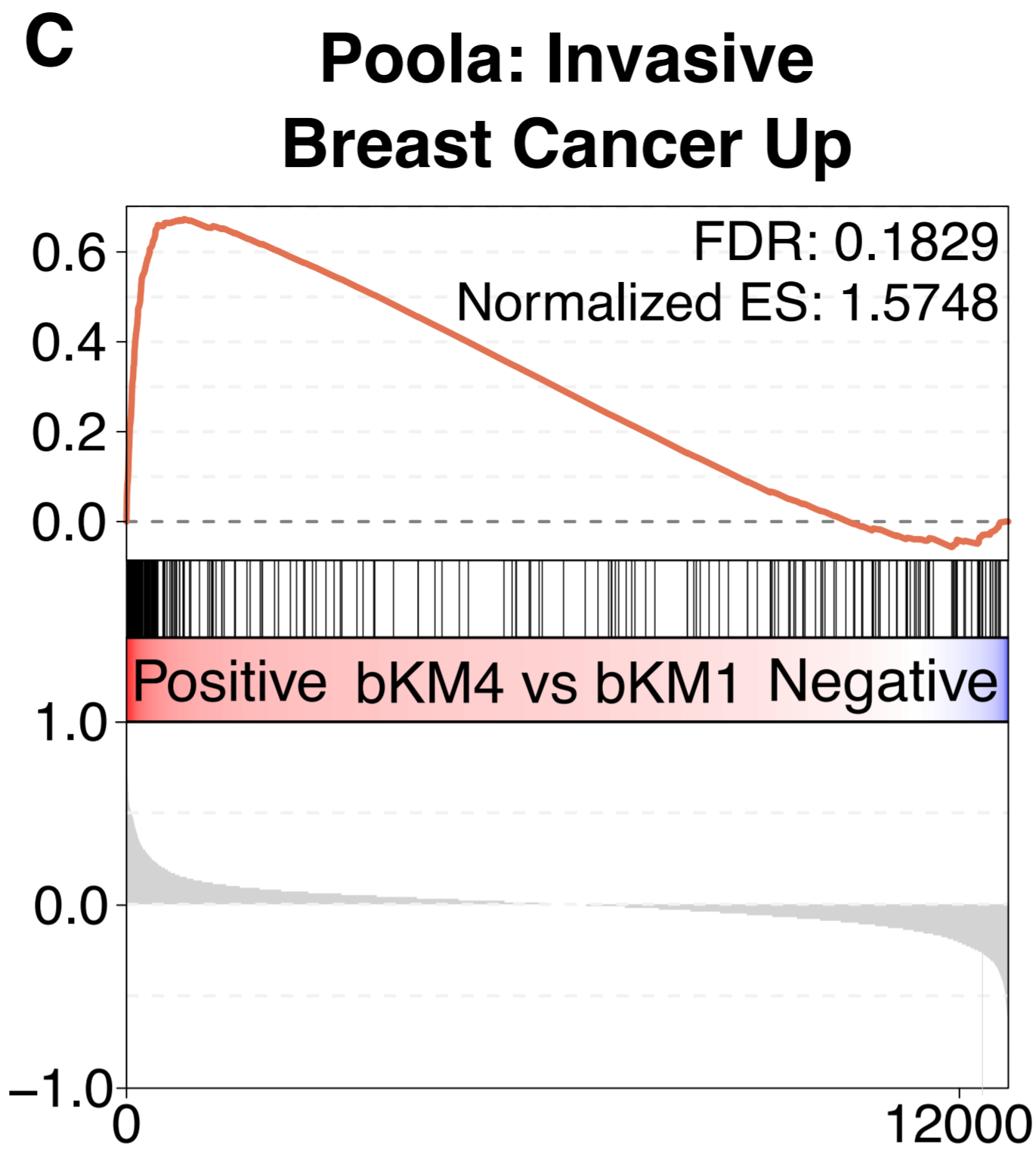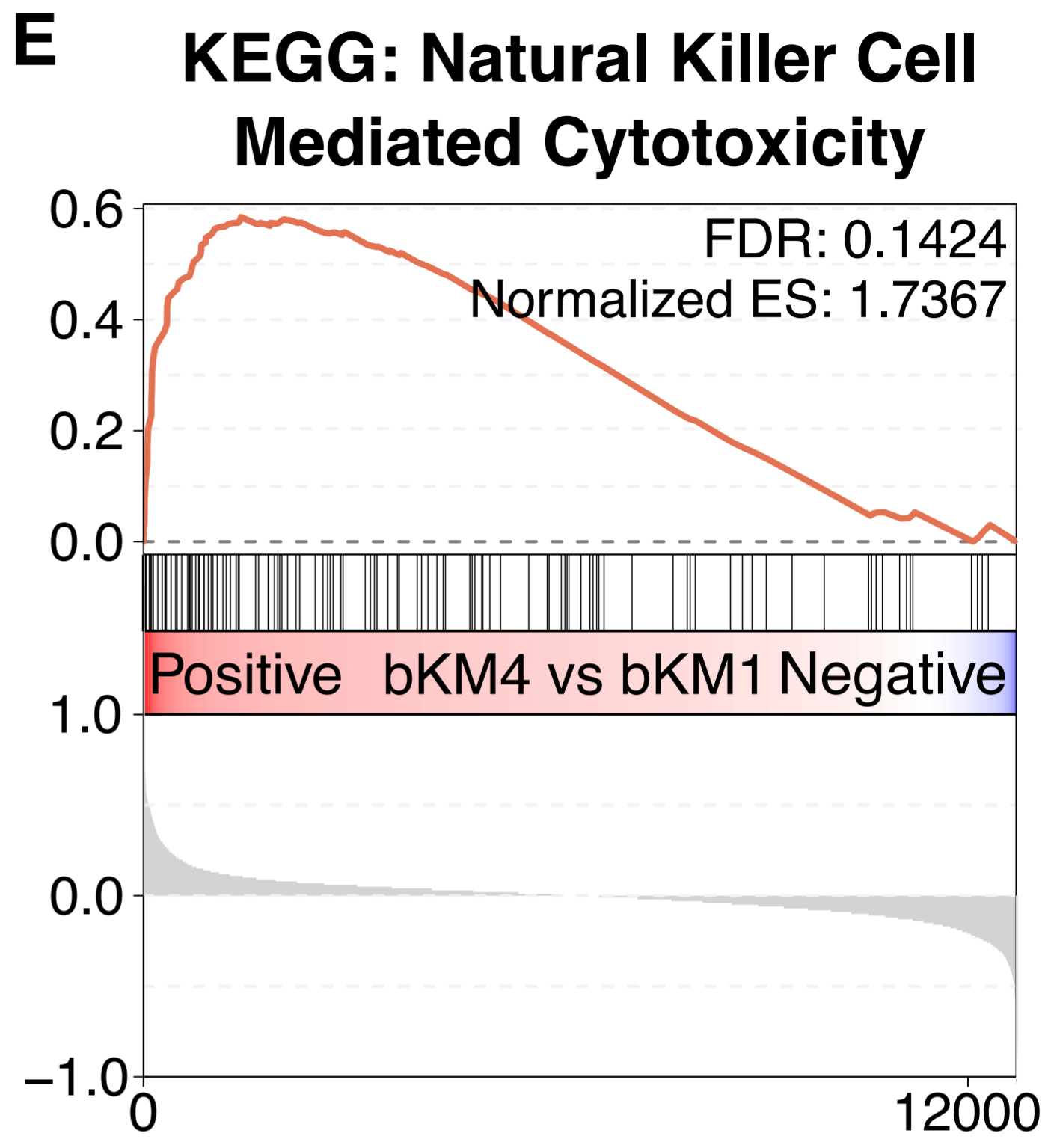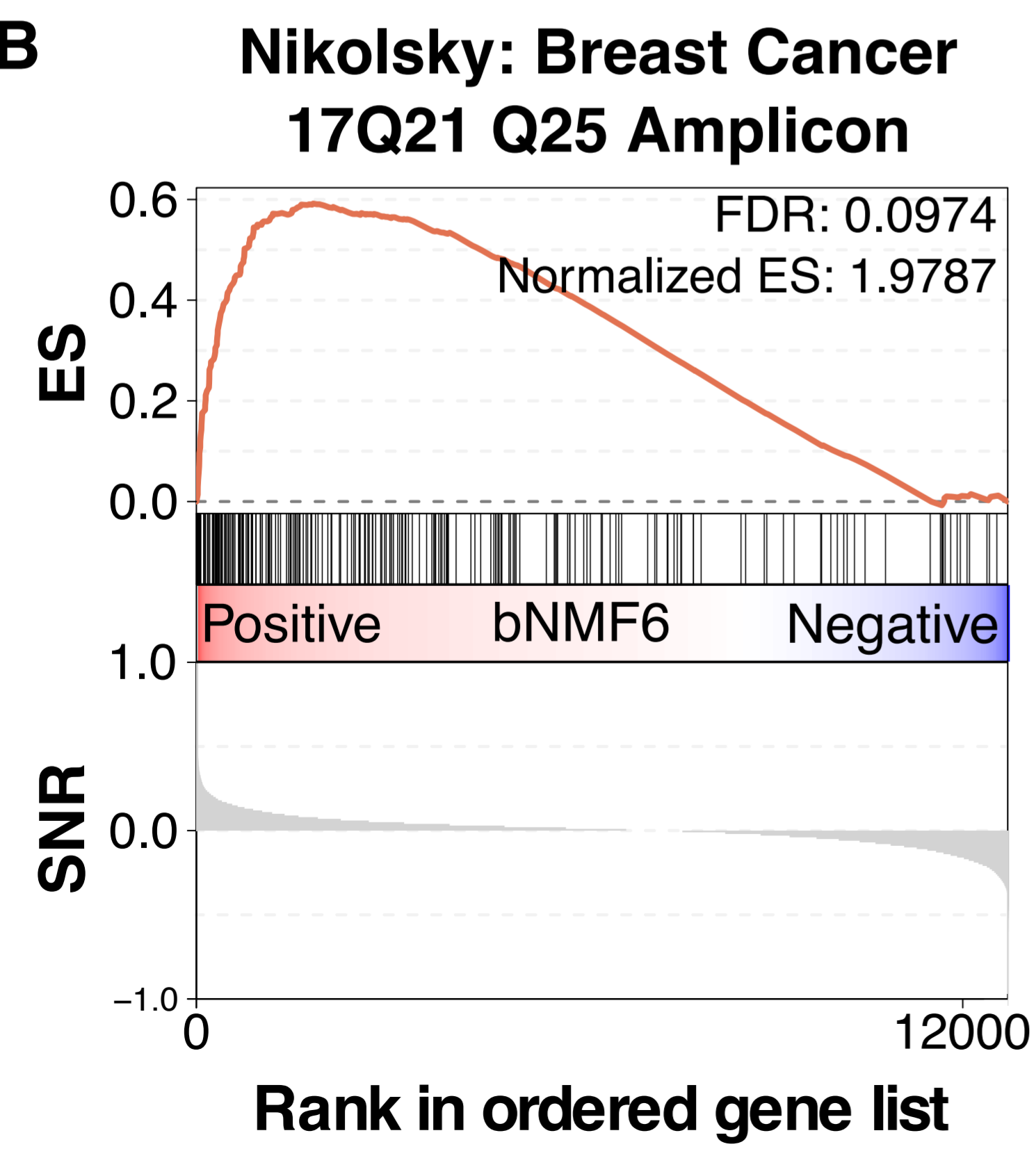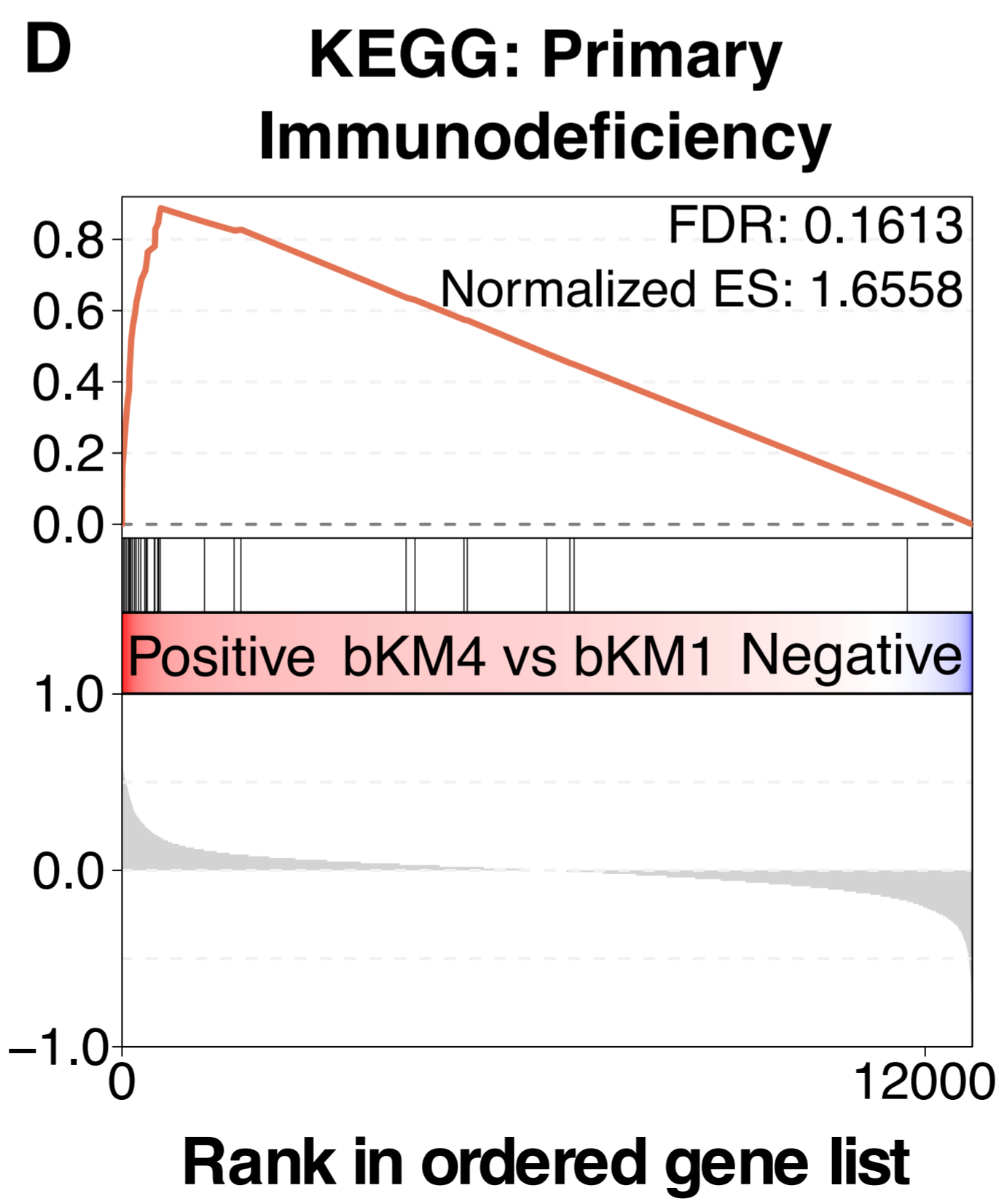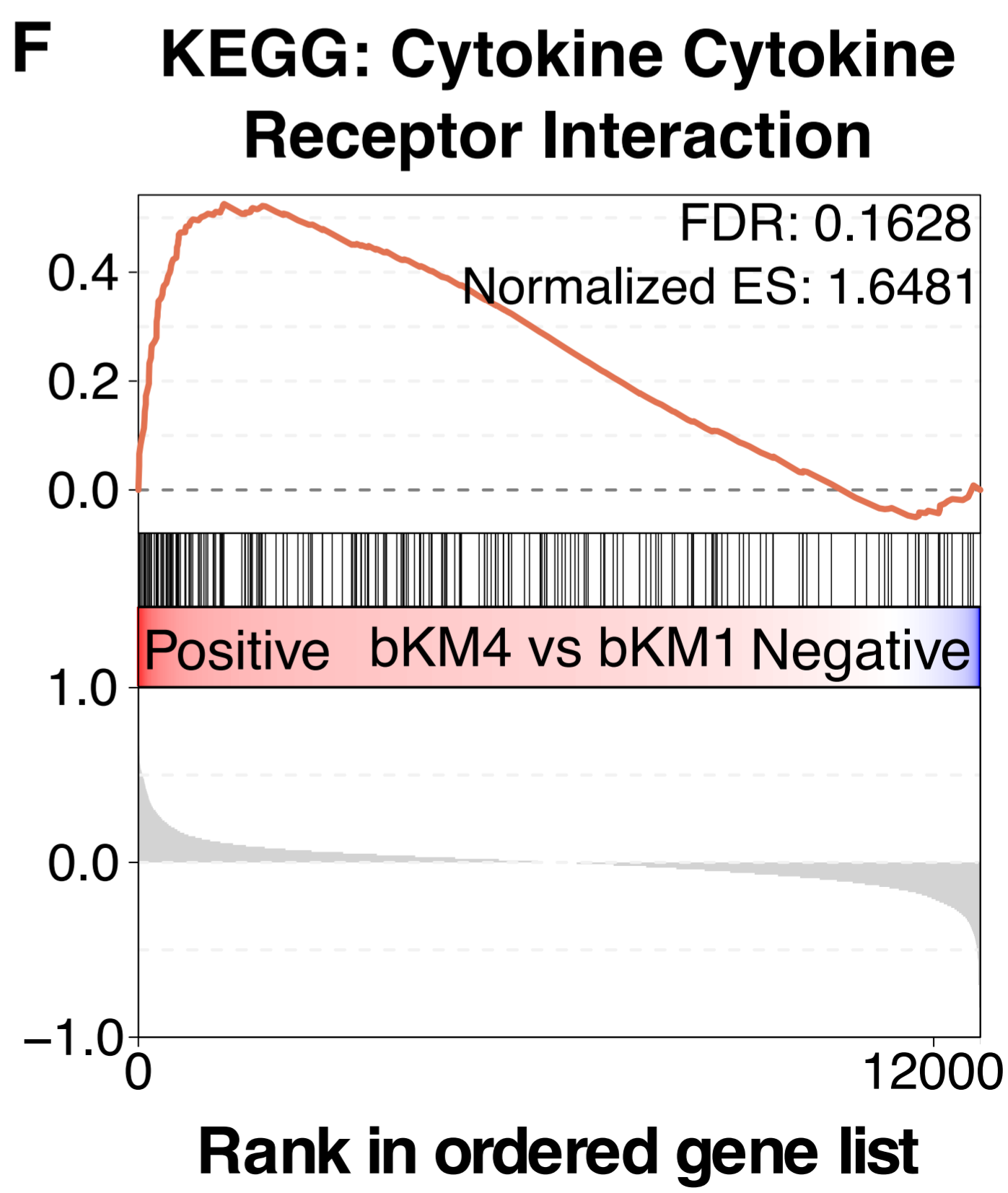

Supplement: Supplementary file 2 — Figure S2. Gene Set Enrichment Analysis (GSEA) analysis between subtypes in breast cancer. (A-F) GSEA analysis between the A) bNMF2 and B) bNMF6 breast cancer clusters, showing gene enrichment of metaplastic breast cancer and 17q21–25 amplicon signatures and C-F) between the two basal-subtype (bKM1 and bKM4) k-means clusters, showing enrichment of invasive and immune-related gene sets in bKM4 cluster. (PDF 119 kb) [file 12859_2018_2204_MOESM2_ESM.pdf]

**Figure S4**

**HYP Reconciliation**

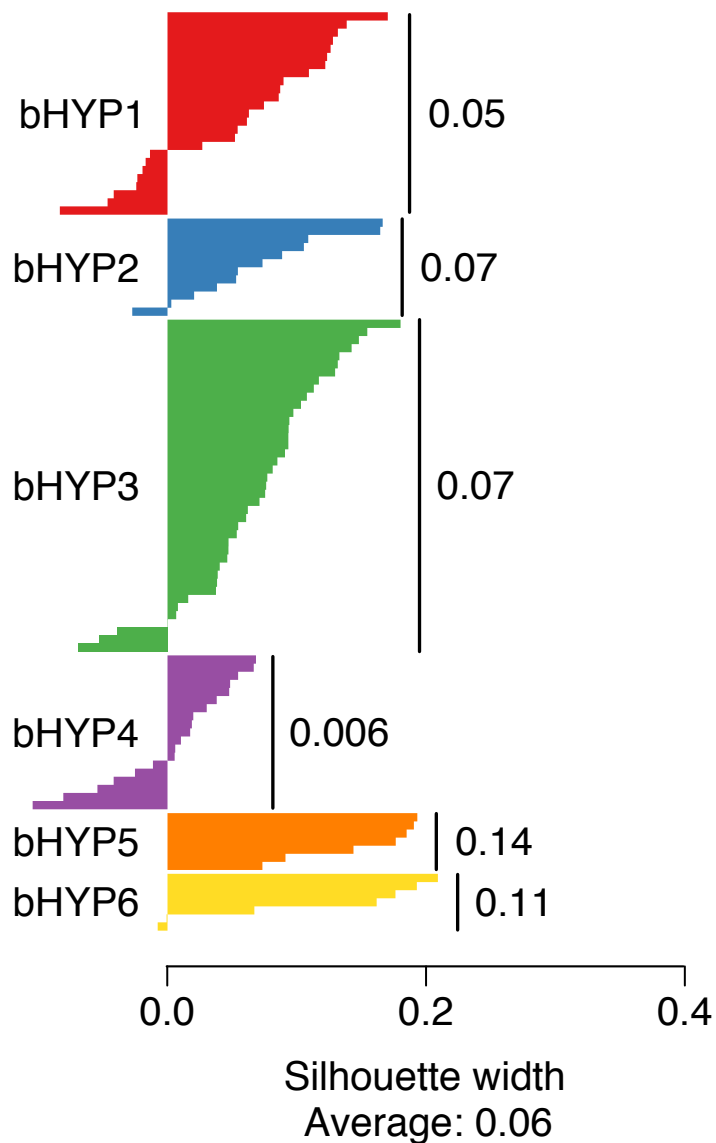

**PMI Reconciliation**

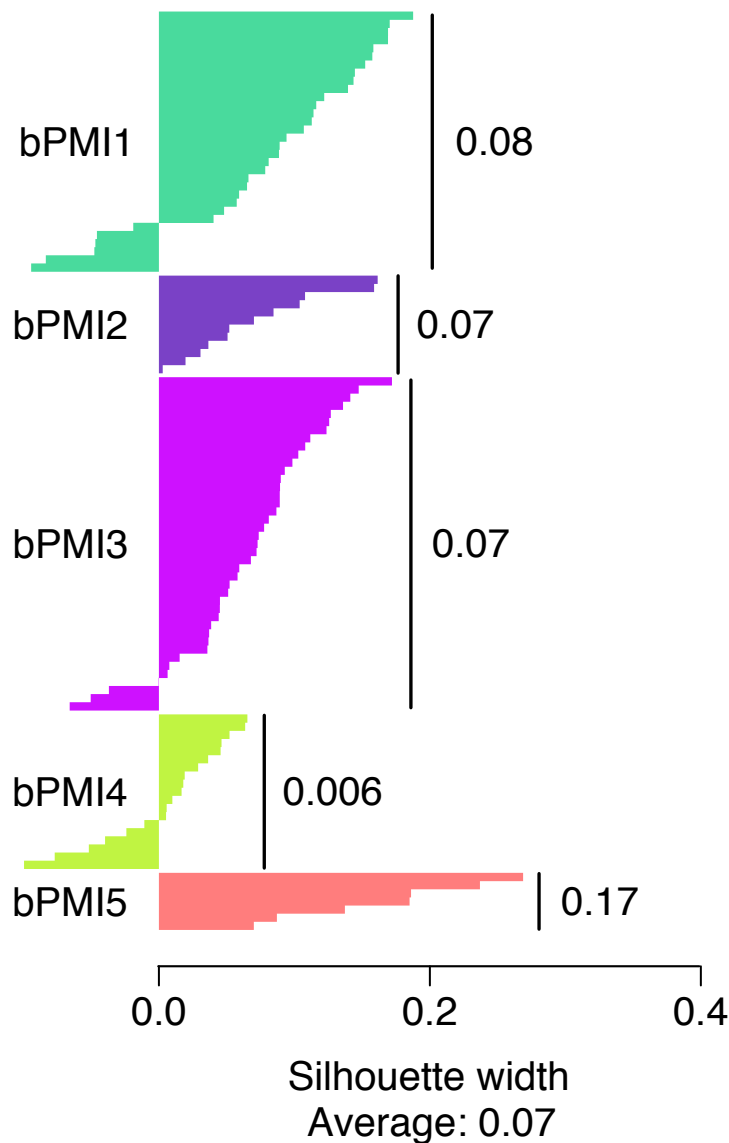

Supplement: Supplementary file 4 — Figure S4. Silhouette width of each sample and community in breast cancer for each reconciliation method –hypergeometric (HYP; left) and PMI (right). Colors represent distinct subtype communities. (PDF 21 kb) [file 12859_2018_2204_MOESM4_ESM.pdf]

Figure S5

A

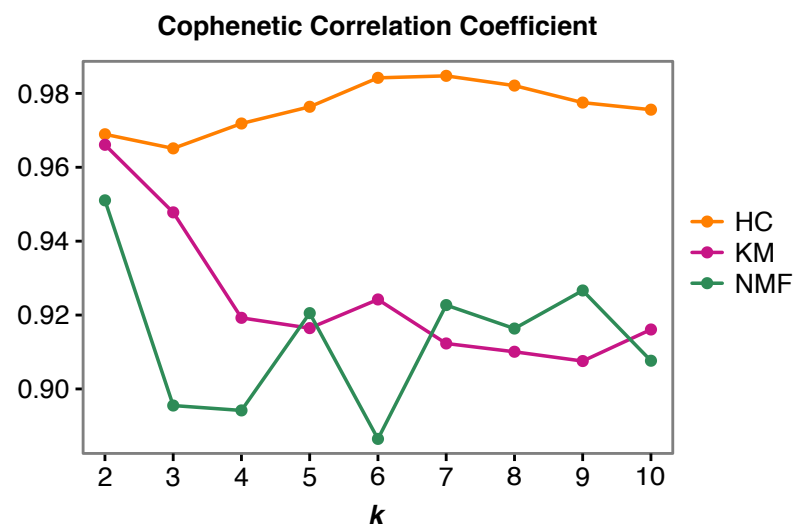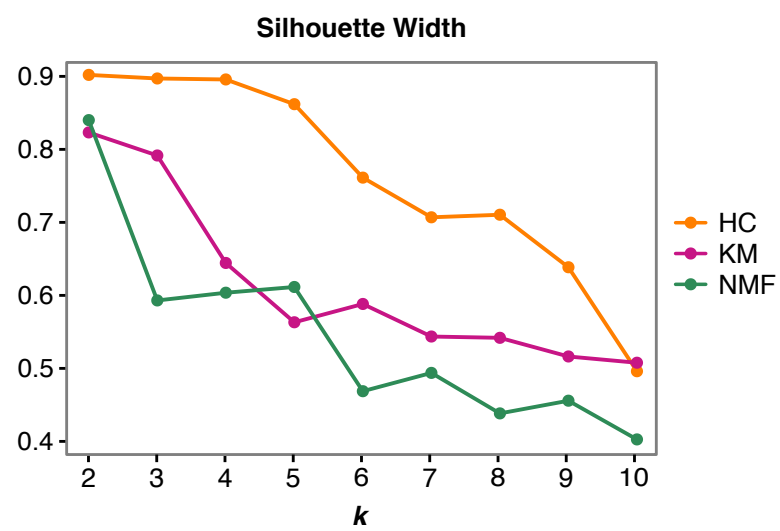

B

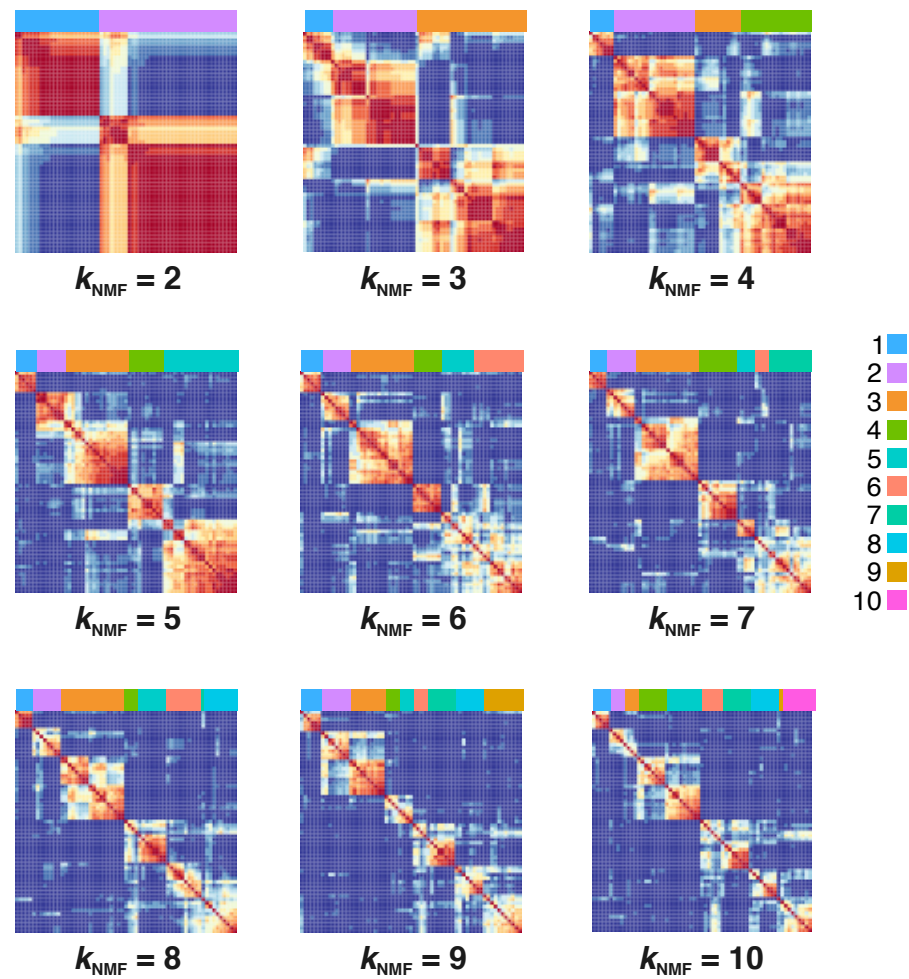

C

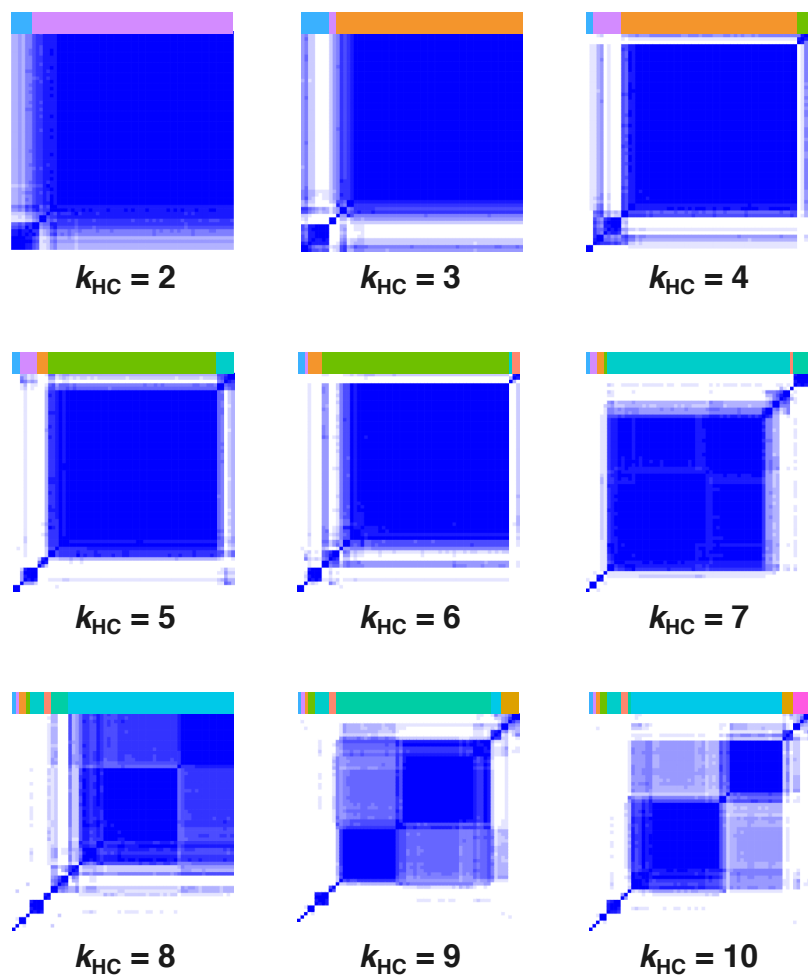

D

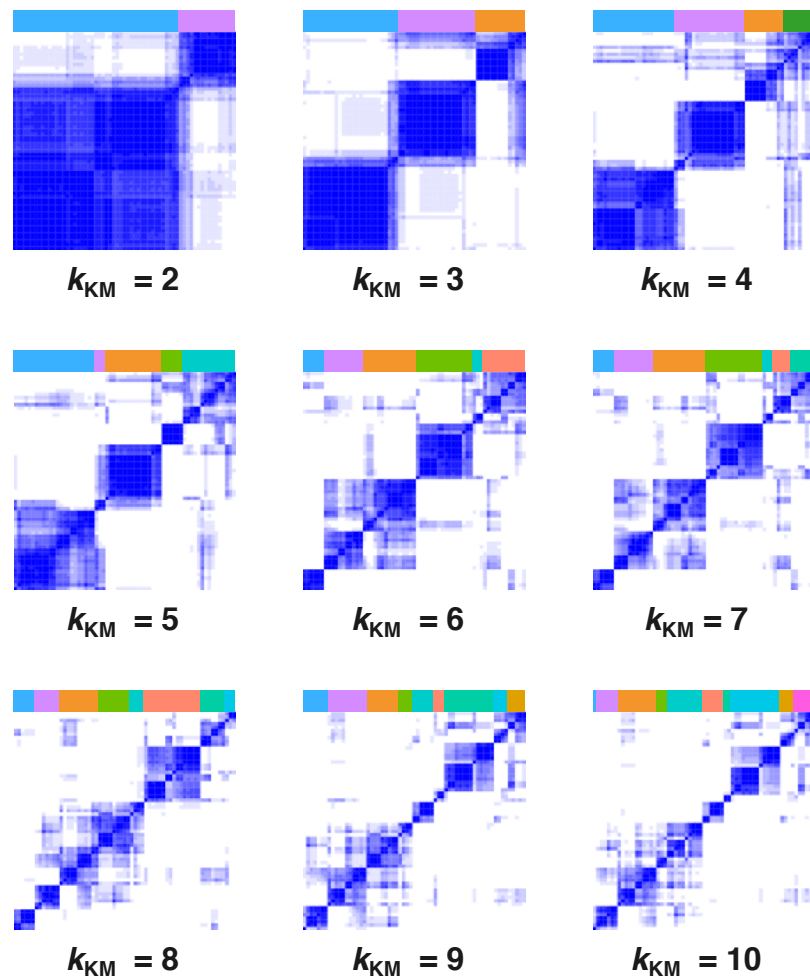

Supplement: Supplementary file 5 — Figure S5. Evaluation of consensus clustering of the uveal melanoma dataset from k = 2 to k = 10. (A) Cophenetic correlation coefficient (upper) and silhouette width (lower) of the clustering generated by each algorithm for each of k clusters. (B) Consensus matrices for NMF clustering. Colors towards red indicate high consensus between different clustering runs and those towards blue indicate low consensus. (C-D) Consensus matrices for C) hierarchical clustering and D) k-means clustering. Blue indicates high consensus between clustering runs and white indicates low consensus. (PDF 467 kb) [file 12859_2018_2204_MOESM5_ESM.pdf]

**Figure S7**

**HYP Reconciliation**

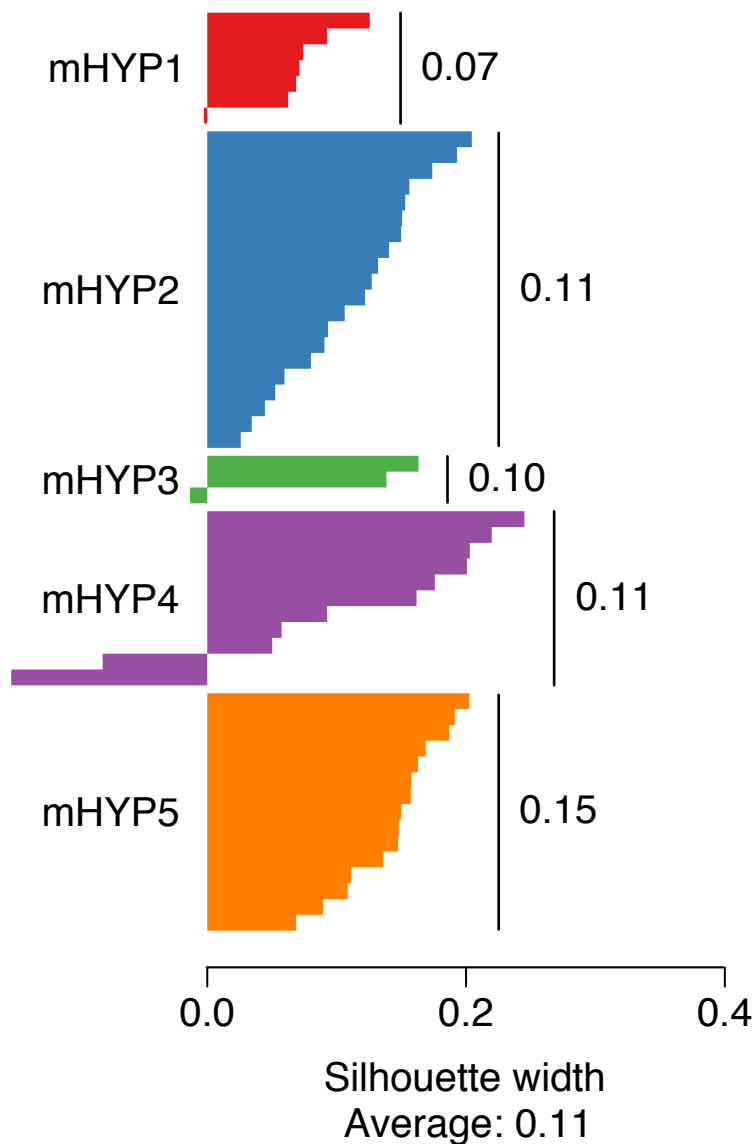

**PMI Reconciliation**

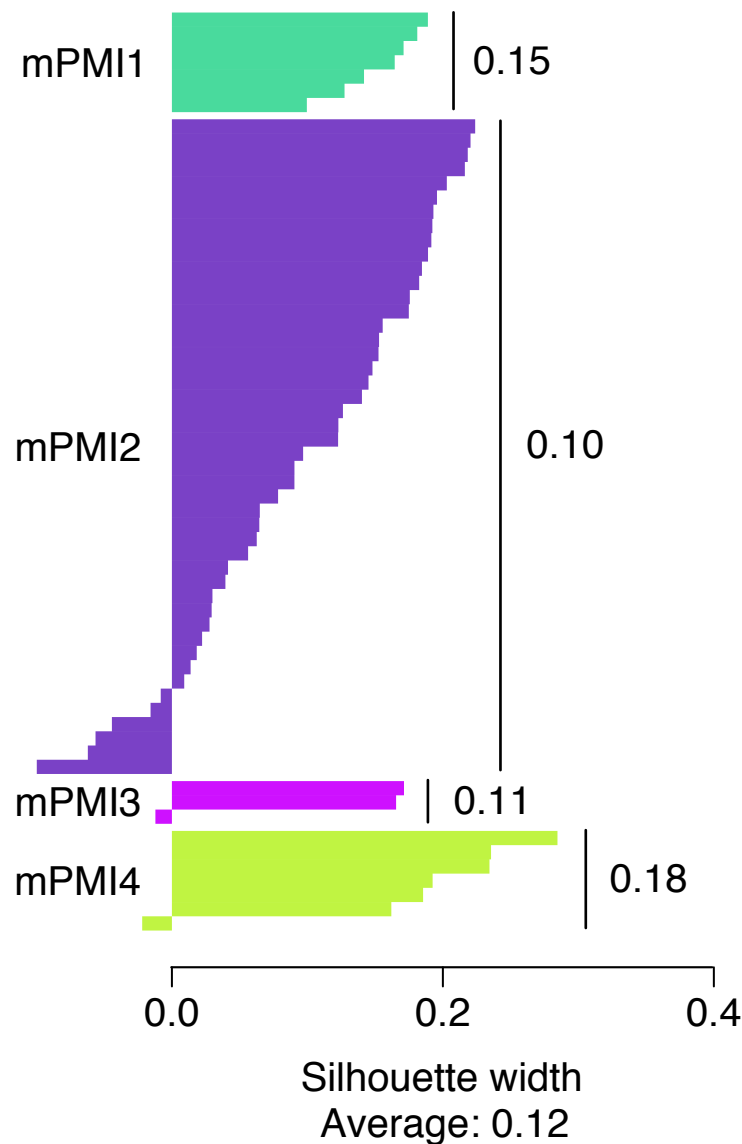

Supplement: Supplementary file 7 — Figure S7. Silhouette width of each sample and community in uveal melanoma for each reconciliation method – hypergeometric (HYP; left) and PMI (right). Colors represent distinct subtype communities. (PDF 19 kb) [file 12859_2018_2204_MOESM7_ESM.pdf]
