# Supplementary material for: polyClustR: defining communities of reconciled cancer subtypes with biological and prognostic significance
Source: BMC Bioinformatics. 2018 May 25;19:182. doi: 10.1186/s12859-018-2204-4 (PMC5970540; doi:10.1186/s12859-018-2204-4)
Supplement: Supplementary file 3 — Figure S3. Comparison of community classifications from each reconciliation method with intrinsic breast cancer subtypes. (A-B) Heatmap showing hypergeometric test with overlap between the subtype communities (from polyClustR) and the known subtypes from A) hypergeometric and B) PMI reconciliation methods. Norm – normal-like subtype; LumA – luminal A subype; Lum B – luminal B subtype. (PDF 50 kb) [file 12859_2018_2204_MOESM3_ESM.pdf]

Figure S3

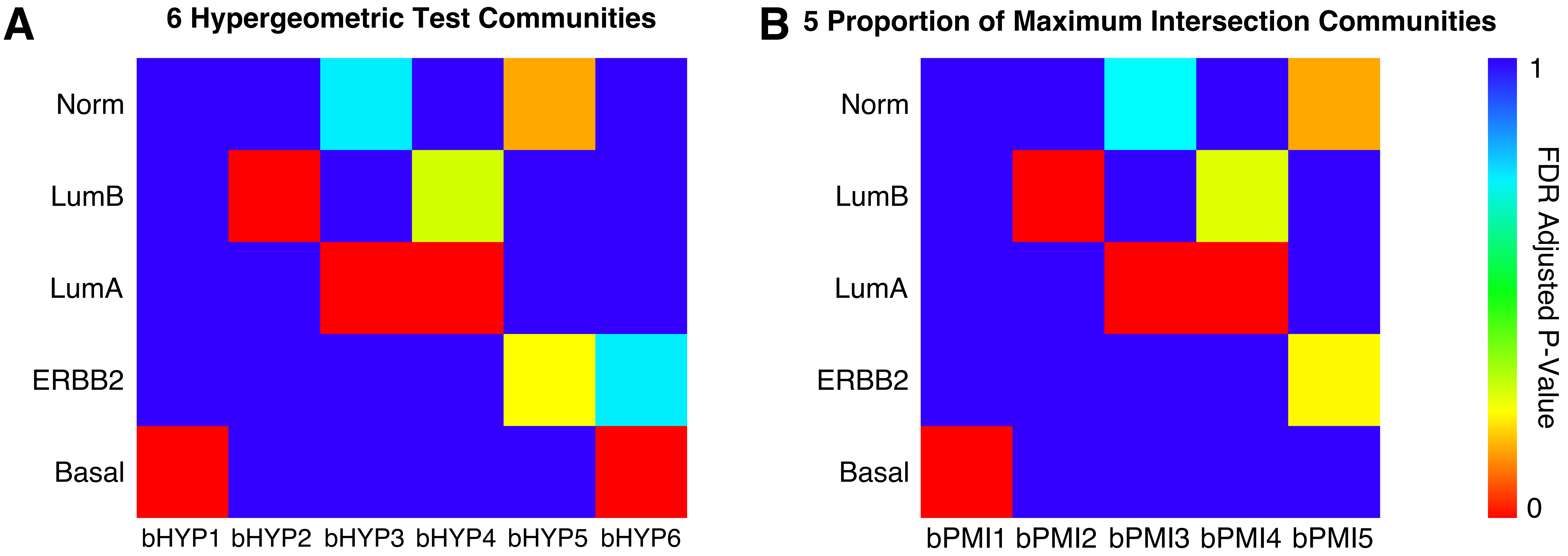
A
6 Hypergeometric Test Communities
Norm
LumB
LumA
ERBB2
Basal
bHYP1 bHYP2 bHYP3 bHYP4 bHYP5 bHYP6
B
5 Proportion of Maximum Intersection Communities
Norm
LumB
LumA
ERBB2
Basal
bPMI1 bPMI2 bPMI3 bPMI4 bPMI5
FDR Adjusted P-Value
1
0
