# Supplementary material for: polyClustR: defining communities of reconciled cancer subtypes with biological and prognostic significance
Source: BMC Bioinformatics. 2018 May 25;19:182. doi: 10.1186/s12859-018-2204-4 (PMC5970540; doi:10.1186/s12859-018-2204-4)
Supplement: Supplementary file 6 — Figure S6. Comparison of community classifications from each reconciliation method with known chromosome 3 ploidy statuses in uveal melanoma. (A-B) Heatmap showing hypergeometric test with overlap between the subtype communities (from polyClustR) and the known ploidy status from A) hypergeometric and B) PMI reconciliation methods. (PDF 53 kb) [file 12859_2018_2204_MOESM6_ESM.pdf]

Figure S6

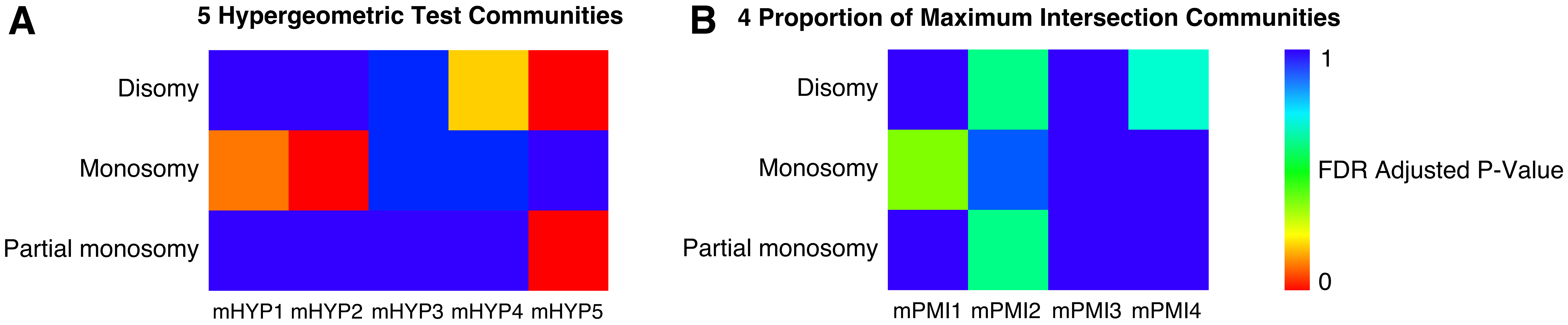
A
5 Hypergeometric Test Communities
Disomy
Monosomy
Partial monosomy
mHYP1 mHYP2 mHYP3 mHYP4 mHYP5
B
4 Proportion of Maximum Intersection Communities
Disomy
Monosomy
Partial monosomy
mPMI1 mPMI2 mPMI3 mPMI4
1
FDR Adjusted P-Value
0
